# Telepsychiatry in the Age of COVID: Some Ethical Considerations

**DOI:** 10.1017/S0963180120000523

**Published:** 2020-06-24

**Authors:** H. PAUL CHIN, GUILLERMO PALCHIK

**Keywords:** telepsychiatry, medical ethics, COVID-19

## Abstract

The COVID-19 pandemic has necessitated a rapid escalation in the use of telepsychiatry. Herein we revisit some of the ethical issues regarding its use, including patient benefice, distributive justice, privacy, and autonomy. Based on these considerations we would hold that telepsychiatry is a vital aspect of providing psychiatric care, and ethically should be offered as a format for treatment, likely beyond the pandemic period. Investigative and advocacy efforts will need to continue to determine its exact role within psychiatric care, and expand its availability for those most in need.

The severe acute respiratory syndrome coronavirus 2 (SARS-CoV-2) pandemic has hastened the widespread deployment of remote access medical care, in line with social distancing guidelines aimed to curb the spread of the virus. For psychiatry, this conversion to telepsychiatry for most has necessarily been swift and broad, particularly for a field stereotypically known for its contemplative and measured ways. In fact, use of telehealth in psychiatry, primarily via real-time interactive video, had been growing steadily^1^ and is one of the highest among medical specialties,^2^ but pre-COVID use was still far short of the tremendous conversion to telepsychiatry witnessed in these last few months. Accurate metrics of this sweeping change, such as scope of adaptation, modalities used, and settings of practice, among others, have yet to emerge. Ethical considerations regarding telepsychiatry have previously been considered prior to COVID-19^3^ ^,^^4^ ^,^^5^; these are perhaps now even more relevant given telepsychiatry’s sudden increased use, and some concepts are explored below, with reflections on our own experience over the past few months, as well as consideration for telepsychiatry’s future role.

Although telepsychiatry has existed for over 50 years,^6^ ^,^^7^ its recent broad use has been the culmination of two factors: the steady improvements in more widely available technologies and the recent sudden easing of regulatory restrictions, including ones over privacy, licensing, and reimbursement as evidenced in U.S. Congressional legislation,^8^ as one regional example. Given its increasing but yet limited use prior to the COVID-19 pandemic,^9^ one might regard telepsychiatry as a mode of care not made widely available until made requisite by a world-engulfing crisis. The existing evidence for the quality of care via telepsychiatry has been favorable^10^ ^,^^11^; if this continues to hold true, telepsychiatry might be considered a treatment that should have been made available to a wider patient population even prior to COVID-19. The main barriers to pre-COVID use of telepsychiatry appear to have been hesitation on the part of the clinician, as well as regulatory restrictions.^12^ ^,^^13^ ^,^^14^ Hence, privacy issues aside (see below), based on the ethical concept of patient benefice, psychiatry, and those that fund it on behalf of the patient, should have an obligation to continue to offer telepsychiatry as a viable means of care post-COVID. Clinician skill and comfort with using telepsychiatry will need to continue and grow, also in the name of benefice, and payors of care must assure its ongoing availability regardless of the course of the pandemic. The latter appears to be the case in some locales,^15^ ^,^^16^ but the absence of certainty appears to be the one constant in the course of this pandemic. Specifically, the devastating financial scar that COVID-19 is expected to leave is considerable; given the historic lack of support mental health care has suffered, it is not inconceivable that a sustained financial downturn may be used to justify reverting to restricting telepsychiatry access, by payors governmental and otherwise. The potential cost savings associated with telepsychiatry may be a considerable individual patient benefit (e.g., reduced commuting to and from treatment and reduced time off of work), and more evidence which demonstrates potential financial incentives for other stakeholders (e.g., reduced need for hospitalization or other acute care) may help its viability^17^ as an ongoing offered health benefit beyond the pandemic. This would be a boon for individual benefice and autonomy, in terms of quality and choice of care, as well as for the financial needs of distributive justice. Likewise, the potential superiority of telepsychiatric care has been suggested, certainly for some conditions, such as autism or severe agoraphobia.^18^ ^,^^19^ Available and developing technology may also change the nature of treatments possible—SMS texting and virtual reality, for example. It remains to be seen whether the promise of these treatments will bear fruit, in the way of well-designed outcome studies, but should any of them indeed prove beneficial, or even superior to live visits, resuming restrictions on telepsychiatry would seem even more untenable, and unfairly limit the choices that ethically should be offered to patients.

As noted above, the suspension of restrictions on telepsychiatry use has greatly facilitated recent wide access. We have seen a variety of readily available video platforms used, in favor of previously existing tools specifically developed for telepsychiatry that offer more secure privacy but, for most, difficult to access. Should telepsychiatry continue its broad use, new regulatory guidelines will need to be implemented to assure patient privacy and quality of care, the latter of which includes proper licensure of eligible clinicians and prevention of fraud; this would obviously need to be balanced with ease of access. It remains the responsibility of clinicians to be apprised of these changes, and, as necessary, adjust their telepsychiatry practice to assure privacy while maintaining high access of use. During the COVID-19 crisis, we have found that privacy requirements may also be logistical; some of our patients have eschewed video visits, being discouraged by lack of privacy in crowded homes during times of restricted social movement (e.g., shelter-in-place or quarantine); similarly, some of our clinicians have moved from their home office back to the clinic, in the name of preserving patient privacy.

One of the key advantages touted of telepsychiatry is its potential for wider access to care, with a potential to bridge gaps in access due to geography or other logistical barriers (e.g., patients in rural areas with inadequate psychiatric care or patients with mobility difficulties). It remains to be seen whether this may actualize into an appreciable difference on the estimated 1.56 billion in the world^20^ lacking care for their mental health conditions, as telepsychiatry during COVID-19 has appeared to be chiefly used to maintain treatment for existing patients. As has been pointed out,^21^ telepsychiatry may remain elusive for those most in need, in particular those with the most persistent and severe mental illnesses, such as schizophrenia, or for any one of our patients otherwise ill able to afford, or navigate, a device capable of a virtual visit. Locally, support for telepsychiatry in the public sector, which cares for the bulk of these patients with the most need, we have found to be spotty and variable; this stands in sharp contrast to some experiences observed in the nonprofit and private sector of healthcare such as ours, which saw the rapid distribution of mobile devices facilitating in-house and ambulatory telepsychiatry in the first wave of response to the pandemic. Hence, the promise of telepsychiatry facilitating distributive justice is not given, and will need active advocacy and effort to advance it above and beyond a mere convenience for those fortunate enough to make use of it.

Within our practice, as social restrictions ease for the time being currently, and live visits become permissible, we have struggled with if and when to reintroduce face-to-face encounters with our patients. Certainly, patients and clinicians alike have expressed preferences, and a universal consensus is, we have found at this point, hardly the case, despite evidence supporting use of telepsychiatry as noted above. In the absence of clear-cut requirements (e.g., a live visit required for administration of a long-acting antipsychotic medication or an immobile patient only accessible by telepsychiatry), what can we tell ourselves and to our patients for a fully informed choice between telepsychiatry and live visits? When is one preferable over the other? Perhaps this leads us to the core question yet unanswered: what, if any, is the unique quality of the live, person-to-person visit over one via video? And, if such a thing exists, is it vital to the provision of care? Or is this consideration merely a manifestation of lingering reluctance to adapt telepsychiatry as previously described? These echo questions pondered currently at large beyond medicine,^22^ ^,^^23^ ^,^^24^ but perhaps is particularly relevant to psychiatry, and others in related therapeutic arts, where the physician–patient (or clinician–client) relationship is uniquely personal and of import in a successful treatment. The nature of this relationship in the ethics of telepsychiatry has been previously cogently explored,^25^ and appropriately characterized as indefinable, yet discountable only via a moral hazard of potentially eroding the dignity of appreciating the patient as a whole. We have had our own experiences, particularly in telepsychiatric applications to our acute inpatient services. An in-person observation of a depressed patient slowly straining out of her bed for interview may be as equally telling as her verbal narrative that follows, if not more. It remains to be seen whether an aptly named "ineffable"^26^ thing as the physician–patient relationship is subject to substantive empiric study. Indeed, telepsychiatry may shift the very nature of the physician–patient relationship.^27^ But widespread use of telepsychiatry during COVID allows us an unprecedented opportunity to scale our investigative power, in an attempt to answer the true interpersonal—and clinical—difference, if any, between live and telepsychiatry visits, and hence facilitate an informed choice for clinicians and patients alike.

Momentum, fueled by patient preference alone, may cement telepsychiatry’s place as a mainstay of treatment regardless of the COVID-19 pandemic. Indeed, its promise to increase access and quality of care may be transformative for the field. Nevertheless, its future development must be accompanied by continued rigorous study of its efficacy, and diligent advocacy to assure safe and secure use in accessing care for all. These efforts would be the manifestations of ongoing adherence to the core concepts within medical ethics, which must continue to tether the changes to and in psychiatry, current and upcoming.

## References

[1] Spivak S, Spivak A, Cullen B, Meuchel J, Johnston D, Chernow R, Green C, Mojtabai R. Telepsychiatry Use in U.S. Mental Health Facilities, 2010–2017. Psychiatric Services 2020;71(2):121–7. doi:10.1176/appi.ps.201900261. 31615370

[2] Chakrabarti S. Usefulness of telepsychiatry: A critical evaluation of videoconferencing-based approaches. World Journal of Psychiatry 2015;5(3):286–304. doi:10.5498/wjp.v5.i3.286. 26425443 PMC4582305

[3] Hyler SE, Gangure DP. Legal and ethical challenges in telepsychiatry. Journal of Psychiatric Practice 2004;10(4):272–6. doi:10.1097/00131746-200407000-00011. 15552552

[4] Stoll J, Müller JA, Trachsel M. Ethical issues in online psychotherapy: A narrative review. Frontiers in Psychiatry 2020;10:993. doi:10.3389/fpsyt.2019.00993. 32116819 PMC7026245

[5] van Wynsberghe A, Gastmans C. Telepsychiatry and the meaning of in-person contact: A preliminary ethical appraisal. Medicine, Health Care and Philosophy 2009;12(4):469–76. doi:10.1007/s11019-009-9214-y. 19629748

[6] Shore J. The evolution and history of telepsychiatry and its impact on psychiatric care: Current implications for psychiatrists and psychiatric organizations. International Review of Psychiatry 2015;27(6):469–75. doi:10.3109/09540261.2015.1072086. 26397182

[7] Von Hafften A. The history of telepsychiatry. American Psychiatric Association toolkit for telepsychiatry; available at https://www.psychiatry.org/psychiatrists/practice/telepsychiatry/toolkit/history-of-telepsychiatry (last accessed 17 June 2020).

[8] Senate Bill 2741—116th Congress. United States—Creating Opportunities Now for Necessary and Effective Care Technologies (CONNECT) for Health Act of 2019; 2019–2020; available at https://www.congress.gov/bill/116th-congress/senate-bill/2741 (accessed 17 June 2020).

[9] See note 2, Chakrabarti 2015, at 286–304.

[10] Hilty DM, Ferrer DC, Parish MB, Johnston B, Callahan EJ, Yellowlees PM. The effectiveness of telemental health: A 2013 review. Telemedicine Journal and E Health 2013;19(6):444–54. doi:10.1089/tmj.2013.0075. 23697504 PMC3662387

[11] See note 2, Chakrabarti 2015, at 286–304.

[12] Hubley S, Lynch SB, Schneck C, Thomas M, Shore J. Review of key telepsychiatry outcomes. World Journal of Psychiatry 2016;6(2):269–82. doi:10.5498/wjp.v6.i2.269. 27354970 PMC4919267

[13] Cowan KE, McKean AJ, Gentry MT, Hilty DM. Barriers to use of telepsychiatry: Clinicians as gatekeepers. Mayo Clinic Proceedings 2019;94(12):2510–23. doi:10.1016/j.mayocp.2019.04.018. 31806104

[14] Brooks E, Turvey C, Augusterfer EF. Provider barriers to telemental health: Obstacles overcome, obstacles remaining. Telemedicine Journal and E-Health 2013;19(6):433–7. doi:10.1089/tmj.2013.0068. 23590176

[15] Wicklund E. Experts weigh in on post-COVID-19 telehealth rules and policies. *mHealth Intelligence*; 2020 June 15; available at https://mhealthintelligence.com/news/experts-weigh-in-on-post-covid-19-telehealth-rules-and-policies (accessed 17 June 2020).

[16] Laura Dyrda Some temporary telehealth provisions will become permanent, CMS chief says. *Becker’s Hospital Review*; 2020 June 2; available at https://www.beckershospitalreview.com/telehealth/some-temporary-telehealth-provisions-will-become-permanent-cms-chief-says.html#:~:text=Some%20temporary%20telehealth%20provisions%20will%20become%20permanent%2C%20CMS%20chief%20says,-Laura%20Dyrda%20(Twitter&text=The%20number%20of%20CMS%20beneficiaries,aim%20to%20continue%20those%20benefits. (accessed 17 June 2020).

[17] See note 2, Chakrabarti 2015, at 286–304.

[18] See note 10, Hilty et al. 2013, at 444–54.

[19] Hilty D. The evidence base. American Psychiatric Association toolkit for telepsychiatry; available at https://www.psychiatry.org/psychiatrists/practice/telepsychiatry/toolkit/evidence-base (accessed 17 June 2020).

[20] Ngui EM, Khasakhala L, Ndetei D, Roberts LW. Mental disorders, health inequalities and ethics: A global perspective. International Review of Psychiatry 2010;22(3):235–44. doi:10.3109/09540261.2010.485273. 20528652 PMC2935265

[21] Frances A. Future of psychiatry in a post-pandemic world. *Psychiatric Times*; 2020 June 4; available at https://psychnews.psychiatryonline.org/doi/10.1176/appi.pn.2020.5b23 (last accessed 12 Jun 2020).

[22] Twitter BD. Square announce work from home forever option: What are the risks? *Forbes*; 2020 May 18; available at https://www.forbes.com/sites/danabrownlee/2020/05/18/twitter-square-announce-work-from-home-forever-optionwhat-are-the-risks/#65658722565f (last accessed 12 Jun 2020).

[23] Molla R. Office work will never be the same. *Vox*; 2020 May 21; available at https://www.vox.com/recode/2020/5/21/21234242/coronavirus-covid-19-remote-work-from-home-office-reopening (accessed 17 June 2020).

[24] Thompson C. What if working from home goes on … forever? *New York Times*; 2020 June 9; available at https://www.nytimes.com/interactive/2020/06/09/magazine/remote-work-covid.html (accessed 17 June 2020).

[25] See note 5, van Wynsberghe, Gastmans 2009, at 469–76. 10.1007/s11019-009-9214-y 19629748

[26] See note 5, van Wynsberghe, Gastmans 2009, at 469–76. 10.1007/s11019-009-9214-y 19629748

[27] Yellowlees P, Richard Chan S, Burke Parish M. The hybrid doctor-patient relationship in the age of technology—Telepsychiatry consultations and the use of virtual space. International Review of Psychiatry 2015;27(6):476–89. doi:10.3109/09540261.2015.1082987. 26493089

